# Identification and validation of metabolism-related genes signature and immune infiltration landscape of rheumatoid arthritis based on machine learning

**DOI:** 10.18632/aging.204714

**Published:** 2023-05-10

**Authors:** Zhaoyang Guo, Yuanye Ma, Yaqing Wang, Hongfei Xiang, Huifei Cui, Zuoran Fan, Youfu Zhu, Dongming Xing, Bohua Chen, Hao Tao, Zhu Guo, Xiaolin Wu

**Affiliations:** 1 Department of Orthopedics, The Affiliated Hospital of Qingdao University, Qingdao 266003, Shandong Province, China; 2 Department of Nephrology, The First Affiliated Hospital of China Medical University, Shenyang 110001, Liaoning Province, China; 3 Cancer Institute, The Affiliated Hospital of Qingdao University, Qingdao University, Qingdao Cancer Institute, Qingdao 266071, Shandong, China; 4 School of Life Sciences, Tsinghua University, Beijing 100084, China

**Keywords:** rheumatoid arthritis, metabolism-related genes, immune infiltration landscape, diagnostic biomarker, machine learning

## Abstract

Rheumatoid arthritis (RA) causes irreversible joint damage, but the pathogenesis is unknown. Therefore, it is crucial to identify diagnostic biomarkers of RA metabolism-related genes (MRGs). This study obtained transcriptome data from healthy individuals (HC) and RA patients from the GEO database. Weighted gene correlation network analysis (WGCNA), the least absolute shrinkage and selection operator (LASSO), and random forest (RF) algorithms were adopted to identify the diagnostic feature biomarker for RA. In addition, biomarkers were verified by qRT-PCR and Western blot analysis. We established a mouse model of collagen-induced arthritis (CIA), which was confirmed by HE staining and bone structure micro-CT analysis, and then further verified the biomarkers by immunofluorescence. *In vitro* NMR analysis was used to analyze and identify possible metabolites. The correlation of diagnostic feature biomarkers and immune cells was performed using the Spearman-rank correlation algorithm. In this study, a total of 434 DE-MRGs were identified. GO and KEGG enrichment analysis indicated that the DE-MRGs were significantly enriched in small molecules, catabolic process, purine metabolism, carbon metabolism, and inositol phosphate metabolism. AKR1C3, MCEE, POLE4, and PFKM were identified through WGCNA, LASSO, and RF algorithms. The nomogram result should have a significant diagnostic capacity of four biomarkers in RA. Immune infiltration landscape analysis revealed a significant difference in immune cells between HC and RA groups. Our findings suggest that AKR1C3, MCEE, POLE4, and PFKM were identified as potential diagnostic feature biomarkers associated with RA’s immune cell infiltrations, providing a new perspective for future research and clinical management of RA.

## INTRODUCTION

Rheumatoid Arthritis (RA) is a common autoimmune illness that affects the synovial membrane of joints, causing persistent inflammation, joint deterioration, and function loss [1]. As the disease advances, joint tissue is continually eroded, leading to irreversible joint degeneration [2]. While the treatment techniques for RA have improved significantly, comprehending the characteristic diagnostic biomarkers is crucial for the clinical management of RA.

Recent studies have reported the role of abnormal metabolism programming in the development of various diseases, including diabetes, hypertension, fatty liver, and cancer [3–6]. Abnormal metabolism programming also plays a significant role in RA. Multiple studies have suggested that disturbances in the metabolism of glucose, glutamine, and lipids may contribute to the development of RA [7]. Qiu et al. identified abnormal metabolism of glucose, amino acids, lactate, and citric acid in the urine of RA patients through metabolomics analysis. He suggested that increased glycolysis, disturbances in the citric acid cycle, oxidative stress, and proteolytic metabolism may be the key characteristics of RA patients [8]. However, the specific metabolic mechanisms that contribute to the clinical management of RA have not yet been fully elucidated.

Immunodeficiency is another critical characteristic of RA [9]. Generally, the synovium is infiltrated by B cells, T cells, and macrophages, leading to the over-proliferation of fibroblast-like synoviocytes (FLS) and the degradation of cartilage and bone [10]. In the pathogenesis of RA, genetic, epigenetic, and environmental factors predispose the host to autoimmunity and, eventually, joint inflammation. Studies have shown that immune cells contributing to joint inflammation include macrophages, dendritic cells, mast cells, neutrophils, and T and B lymphocytes. These immune cells play a crucial role in the early stages of the disease [11]. However, recent studies have reported that metabolism could regulate the function of immune cells. T cell differentiation and functionality are highly dependent on metabolic adaptation, with significantly different metabolic programming in different functional states [12]. Therefore, exploring the relationship between metabolism and immunity appears to be essential for the future of RA treatment.

Currently, there is considerable attention towards identifying disease-specific biomarkers and utilizing bioinformatics and genome sequencing technologies [13]. Several bioinformatic techniques were used in this work to evaluate the involvement of metabolism-related genes (MRGs) in the development of RA. As a consequence, four MRGs (AKR1C3, MCEE, POLE4, and PFKM) were discovered and validated *in vitro* and *in vivo* as diagnostic feature biomarkers for RA. Moreover, the correlation between these diagnostic feature markers and the immune infiltration landscape of the healthy control (HC) and RA group was evaluated.

## MATERIALS AND METHODS

### Dataset download from GEO database

In this study, we obtained transcriptomic data for both rheumatoid arthritis (RA) and normal samples from the GSE93272 dataset in the GEO database. The data was generated using the [HG-U133_Plus_2] Affymetrix Human Genome U133 Plus 2.0 Array (GPL570). From the GSE93272 dataset, we extracted transcriptomic data for a total of 43 normal and 232 RA samples. A perl script was used to collate and annotate the transcriptomic data for each sample. Additionally, we extracted clinical information for each sample from the matrix file using a perl script.

### MRGs identification and WGCNA analysis

Based on the MSigDB database, we extracted 854 differentially expressed MRGs for further analysis using a Perl script and the reference gene set file “C2.cp.kegg.v7.5.1.symbols.gmt.” We applied the “limma” R package with a false discovery rate (FDR) threshold of < 0.05 to identify significant MRGs. To identify MRGs associated with rheumatoid arthritis (RA), we developed a WGCNA model using the “WGCNA” script. Initially, we performed cluster analysis on each sample to remove outliers and constructed an unscaled network based on the optimal soft threshold. Next, we used dynamic tree cutting and merging of gene modules and calculated the correlation between each gene module and clinical features using the Pearson algorithm. Finally, we selected the most significantly correlated gene module based on the module-trait correlation heatmap for further investigation.

### Machine learning and biomarker screening

To identify potential biomarkers for rheumatoid arthritis (RA), we performed modeling analyses on the MRGs using two different machine learning algorithms. First, we used the “glmnet” R script to select MRGs based on the optimal coefficients and lambda values. Additionally, we used the “randomForest” R script to calculate the importance of each MRG, with a threshold set at greater than 1. We used the “venn” script to identify the intersection of the MRGs selected by WGCNA, RF, and LASSO as potential biomarkers for RA. Based on the expression of these biomarkers, we constructed a nomogram model using the “rms” script to evaluate their diagnostic effectiveness. Finally, we evaluated the AUC values of the biomarkers and nomogram using the “pROC” script.

### Functional enrichment and immune infiltration landscape analysis

In this study, we explored the potential biological functions of differentially expressed MRGs in HC and RA groups using the “clusterProfiler” script. Additionally, we used the Metascape database to explore the biological functions of key gene modules identified through WGCNA. Using the single sample Gene Set Enrichment Analysis (ssGSEA) method and 23 immune cell markers, we assessed the immune infiltration characteristics of HC and RA samples. Using the “GSVA” script, we estimated the percentages of these 23 immune cells for each sample based on their transcriptomic data. We generated a PCA plot using the “ggplot2” script to visually demonstrate dissimilarities in immunological cell infiltration patterns between the two subject cohorts. The correlation between the 23 immune cell types was calculated using the Pearson algorithm.

### Quantitative real-time PCR analysis (qRT- PCR)

We use qRT- PCR to validate diagnostic biomarkers. RNA was extracted from RA patients and healthy controls using TRIzol reagent (Cat# 15596018, Thermo), and then reverse transcribed into cDNA using the PrimeScript™ RT reagent Kit with gDNA Eraser (Perfect Real Time) (Cat# RR047A, Takara). The reverse transcribed cDNA was then subjected to real-time fluorescence quantitative PCR detection using the SYBR Premix Ex Taq II kit (Cat# RR820B, Takara). We used GAPDH as an internal control, and the gene primer sequences used in this experiment were listed in Table 1. Amplification reaction was performed using PCR instrument, and the final results were obtained using the 2(-ΔΔCt) method to analyze the RNA expression level of the corresponding samples in this experiment.

**Table 1:** The primers used for qPCR detection.

**Gene name**	**Forward**	**Reverse**
AKR1C3	TAATGAGGAGCAGGTTGGACT	CAACTCTGGTCGATGAAAAGTGG
MCEE	ACATCACAGCCCTTGGATCA	AGGGACCGCTTCACTTACCT
POLE4	GCCATCTTCATTCTGGCACG	CCAAGTCTCTCCTCTGAAGGG
PFKM	CCACTGTGAGGATTGGCCTT	CAGTCCAGCCCCCAACATAG
GAPDH	CATGTTCGTCATGGGTGTGAA	GGCATGGACTGTGGTCATGAG

### Western blot analysis

We use Western blot analysis to validate diagnostic biomarkers. Tissue extracts from RA patients and control groups were lysed using radioimmunoprecipitation (RIPA) buffer (high) (Cat#R0010, Solarbio). Then, we determined the protein concentration using the BCA Protein Assay Kit (Cat#PC0020, Solarbio) according to the instructions provided in the kit. The required protein samples were separated using 10% or 15% Sodium Dodecyl Sulfate-Polyacrylamide Gel Electrophoresis (SDS-PAGE) and transferred onto 0.22μm Polyvinylidene Fluoride (PVDF) membranes (Cat#ISEQ00010, Millipore). After blocking with 5% skimmed milk powder at room temperature for 1 hour, the membrane was incubated overnight in the desired detection solution (Table 2) at 4° C. On the second day, the incubated membrane was removed and incubated in a secondary antibody solution labeled with horseradish peroxidase (HRP) (Elabscience, China) for 1 hour. Then, chemical luminescence was performed using the Super Excellent Chemiluminescent Substrate (ECL) Detection Kit (Cat#E-IR-R308, Elabscience), and the membrane was exposed in an imaging device (Odyssey® XF). The resulting image was then analyzed.

**Table 2:** Primary antibodies and IgG controls used in this study.

Antibody*	**Host**	**Supplier/catalog no.**	**Dilution**
AKR1C3	Rabbit polyclonal	Abcam/ab209899	1:1000(Wb) 1:100(IHC)
MCEE	Mouse monoclonal	Abcam/ab236397	1:2000(Wb)
MCEE	Rabbit polyclonal	Biorbyt/orb27886	1:100(IHC)
POLE4	Rabbit polyclonal	Solarbio/K006102P	1:1000(Wb)
POLE4	Rabbit polyclonal	Biorbyt/orb674262	1:300
PFKM	Rabbit polyclonal	Abcam/ab154804	1:1000(Wb) 1:100(IHC)
GAPDH	Mouse monoclonal	Proteintech/60004-1-Ig	1:50000(Wb)
IgG control	Mouse	Elabscience/E-AB-1001	1:2000(Wb)
IgG control	Rabbit	Elabscience/E-AB-1003	1:5000(Wb)
IgG control	Rabbit	Elabscience/E-AB-1010	1:100(IHC)

### Animals

A total of 40 male Balb/c mice (6-8 weeks old) were utilized in this investigation. Mice were allowed to eat and drink ad libitum and housed in an environmentally controlled room. The experimental animals were taken from the Shandong Province’s Qingdao University Experimental Animal Center.

### Clinical specimens

Between August 2021 and October 2022, we collected 8 meniscus tissue samples from human RA patients at Qingdao University Affiliated Hospital who met the clinical and radiographic diagnostic criteria for RA. Eight additional meniscus tissue samples were collected from young patients requiring surgical repair of meniscus tears, who served as a control group and had no history of RA.

### Experimental grouping

Forty male Balb/c mice aged 6-8 weeks were used in this study and randomly divided into two groups: the Sham group and the Collagen-induced arthritis (CIA) group. The groups were defined as follows: The CIA group is widely used as an animal model since it shares many pathological and immune features with human rheumatoid arthritis (RA) [14]. The CIA model in DBA1/J mice has been extensively employed in RA research [15, 16]. In our study, we established the CIA mouse model following the protocol described by David et al. [17]. On day 0, we injected 2 mg/mL bovine type II collagen combined with an equivalent amount of complete Freund’s adjuvant (CFA) (total 100μl) into the mice’s tail vein. To boost the immunological response, we injected 2 mg/mL of bovine type II collagen mixed with an equivalent amount of incomplete Freund’s adjuvant (IFA) into the tail vein on day 21. In contrast, the Sham group mice were injected with 100 μl of saline on day 0 and day 21, and the other procedures were the same as those in the CIA group. We monitored the progression of arthritis in the mice daily.

### HE staining

To confirm the success of mouse modeling, we utilized hematoxylin-eosin (HE) staining. After euthanizing the mice, their hind limbs were dissected and fixed in 10% neutral-buffered formalin for 2 days. Following this, the specimens were decalcified, embedded in paraffin, and sectioned. The paraffin blocks were then deparaffinized in xylene, gradually dehydrated in ethanol, and stained with hematoxylin-eosin. The sections were differentiated and counterstained with eosin. Following the staining process, the sections were dehydrated in gradient ethanol and mounted with neutral resin. The specimens were then observed under a light microscope to confirm the successful modeling of mice.

### Micro-CT validation analysis of bone morphology and structure

Micro-CT is a commonly used imaging technique for the visualization and analysis of bone and joint structures in small animals such as mice. It uses X-rays to create high-resolution 3D images of the internal structure of an object. In this study, micro-CT was used to verify the success of the modeling of mice by examining the knee joints of the hind limbs. The Quantum GX2 microCT imaging system from PerkinElmer was used to acquire CT image sets for 4 minutes, using specific beam parameters and an X-ray filter to optimize image quality. The resulting images were then analyzed to assess changes in bone and joint structures between the sham and CIA groups.

### Immunofluorescence validation experiment

Immunofluorescence was utilized to validate diagnostic biomarkers in this study. Mouse joint sections were deparaffinized and treated with 10 mM citrate buffer (Elabscience). The sections were then blocked with 5% BSA (Solarbio, China) for 1 hour before incubation with primary antibody solution (diluted according to the antibody datasheet) overnight at 4° C. The next day, the incubated sections were retrieved and subjected to a 1-hour incubation with a fluorescence-labeled secondary antibody solution (provided by Elabscience), which corresponded to the specific antibodies used in the experiment as listed in Table 2. The sections were washed three times with 1×TBST solution for 10 minutes after each incubation. DAPI (blue) was used for nuclear counterstaining with a fluorescence dye, and an anti-fluorescence quencher was added to seal the cover glass. The samples were observed and imaged using a confocal microscope (NIKON, Japan).

### Measurement and analysis of 2D TOCSY spectra by NMR of human meniscus *ex vivo* tissue

We utilized NMR to perform a metabolite analysis on the RA and HC groups. The human knee joint tissue was first ground and homogenized in a mixture of dichloromethane and methanol (V dichloromethane: V methanol = 3:1) and then subjected to high-speed and low-temperature homogenization (4° C, 15000g). Bruker AVANCE 400MHz nuclear magnetic resonance (NMR) (Bruker® Corporation, Germany) was used to obtain the data, with the following parameters: Pulse Sequence: mlevphpp, Probe: Z163739_0119 (PI HR-400-S1-BBF/H/D-5.0-Z SP), Number of Scans: 4, Receiver Gain: 21.0MHz, Relaxation Delay: 1.8976ms, Acquisition Time: 0.3543ms, Spectrometer Frequency: 400.15MHz, Solvent: D2O, SN> 340mm 1x 90° pulse <10, linear <0.6pm 5pm 9 (rotation). The data was then processed using Mestrenova 14.0.0 (Mestrelab Research® Corporation, Spain), which included spectral baseline correction, Fourier transformation, F1 and F2 phase correction, signal suppression processing, and window and zero function settings. The lactate (Lac) peak was calibrated, and after peak labeling, TOCSY 2D spectra were created to infer and compare potential compound types with changes in cartilage metabolite compound types between the RA group and the normal group.

### Statistical analysis

We performed statistical and data visualization analyses using Perl scripts, R software version 4.1.0, and GraphPad Prism version 8.0.1. The potential correlation between biomarkers and immune infiltration was evaluated using Spearman’s rank correlation algorithm. T-test analysis was used to evaluate the statistical differences between two groups, and a p-value < 0.05 was regarded statistically significant.

### Data availability statement

All data and clinical information involved in this paper were obtained from a public database, approved from the Ethics committee and written informed consent from patients were not required.

## RESULTS

### Differential expression of metabolism-related genes (DE-MRGs) selection and functional enrichment analyses

**Figure 1:**
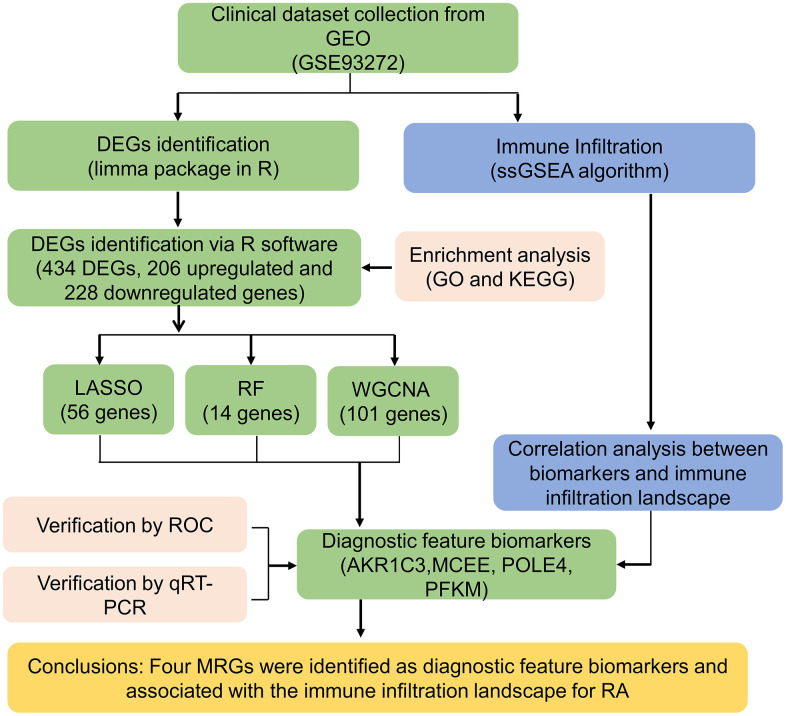
The workflow of the analysis process.

**Figure 2:**
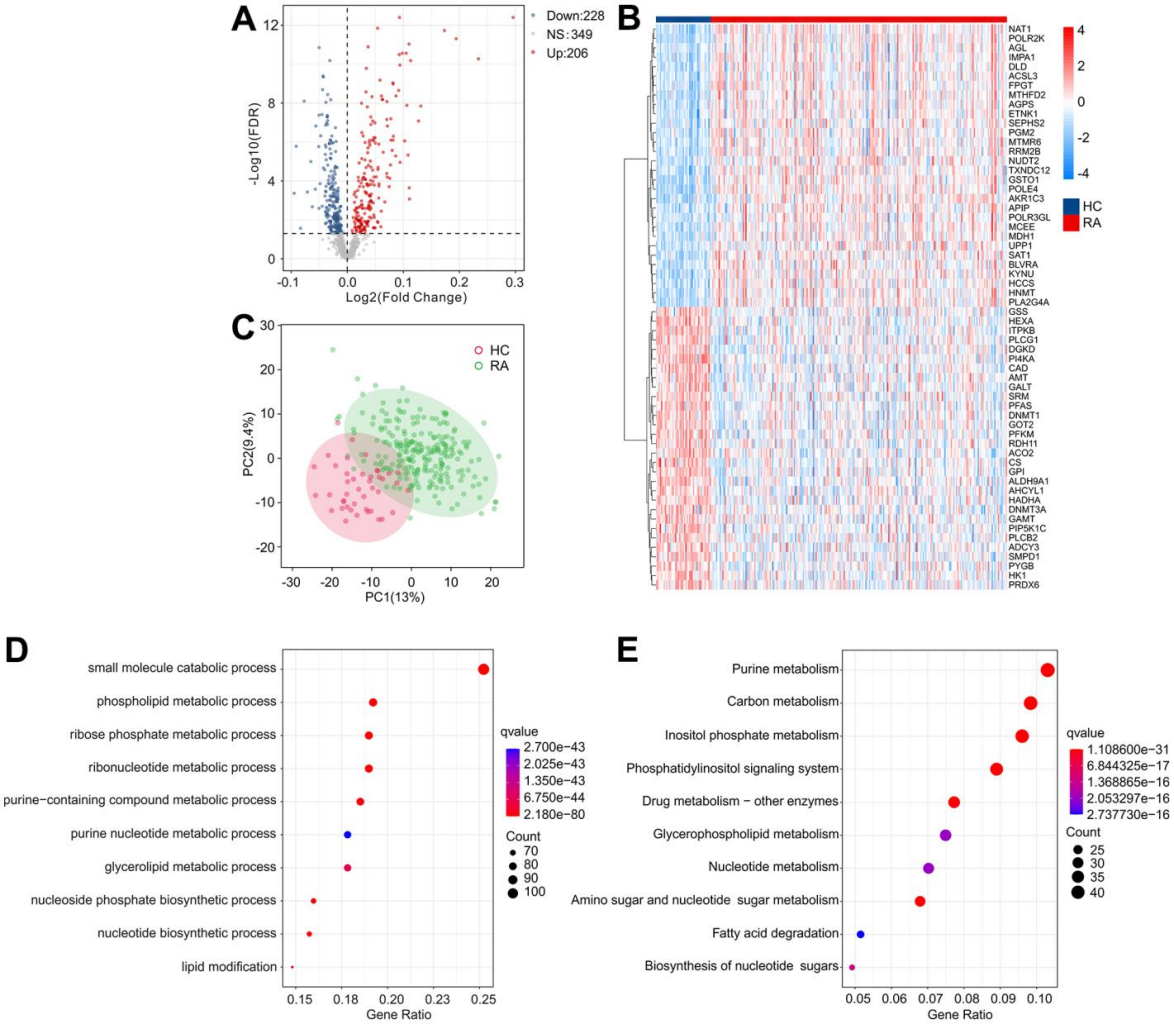
**Identification of DE-MRGs and functional enrichment analyses.** (**A**) Volcano diagram shows the DE-MRGs with the threshold setting at |Fold change| ≥ 1 and FDR < 0.05. Red dots represent upregulated differential genes, gray dots represent no significant difference, and blue dots represent down-regulated genes. (**B**) The expression of top 30 upregulated and down-regulated differential genes in HC and RA groups. (**C**) Principal component analysis shows a significant separation between HC and RA groups based on the MRGs. The top 10 enrichment results of (**D**) Gene Ontology (GO) and (**E**) Kyoto Encyclopedia of Genes and Genomes (KEGG) signaling pathway based on DE-MRGs.

The workflow of this research is depicted in Figure 1. A cut-off value of p(FDR) < 0.05 was set, and the “limma” script was used to calculate differentially expressed MRGs between 43 HC and 232 RA samples. As shown in Figure 2A, 2B, a total of 434 MRGs were identified as DE-MRGs, comprising 206 upregulated genes and 228 downregulated genes. The PCA results indicated that MRG expression could accurately distinguish between HC and RA samples, demonstrating significant differences in MRGs between the two groups (Figure 2C). The GO analysis revealed that the DE-MRGs were associated with ribose phosphate metabolic processes, phospholipid metabolic processes, and small molecule catabolic processes, while the KEGG results showed that DE-MRGs were closely related to metabolic pathways such as purine metabolism, carbon metabolism, and inositol phosphate metabolism (Figure 2D, 2E).

### Development of weighted gene co-expression network analysis (WGCNA)

**Figure 3:**
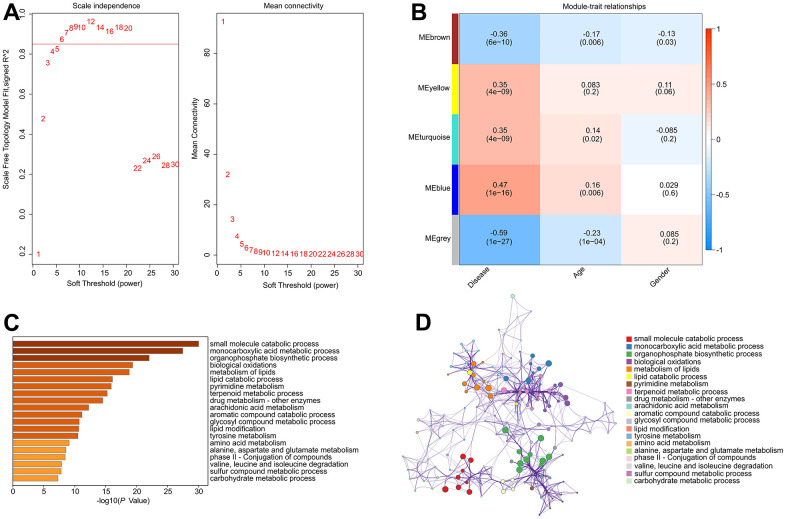
**Construct weighted gene co-expression network analysis based on the DE-MRGs.** (**A**) Analysis of the scale-free network for various soft-thresholding powers (β). (**B**) Heatmap shows the correlation of module eigengenes and clinical characteristics. (**C**, **D**) Functional enrichment analysis of genes in module blue.

To investigate the critical DE-MRGs in the development of RA, a gene co-expression network was constructed by WGCNA. The power of β = 6 was set as the soft-threshold parameter to construct a scale-free network with a scale-free R2 value greater than 0.85. The module eigengenes and clinical characteristics correlation coefficient were then computed (Figure 3A). The module trait relationships results revealed a significant correlation between multiple module eigengenes and clinical characteristics (Figure 3B). Module blue was observed to be positively correlated with RA so that was selected for subsequent analysis (R2 = -0.47, p = 1e-16). The results of functional enrichment analysis revealed that the genes in module blue were primarily involved in the organophosphate biosynthetic process, monocarboxylic acid metabolic process, small molecule catabolic process and biological oxidations, indicating a potential impact of DE-MRGs on molecular mechanism in the development of RA (Figure 3C, 3D).

### Identification of diagnostic feature biomarkers

**Figure 4:**
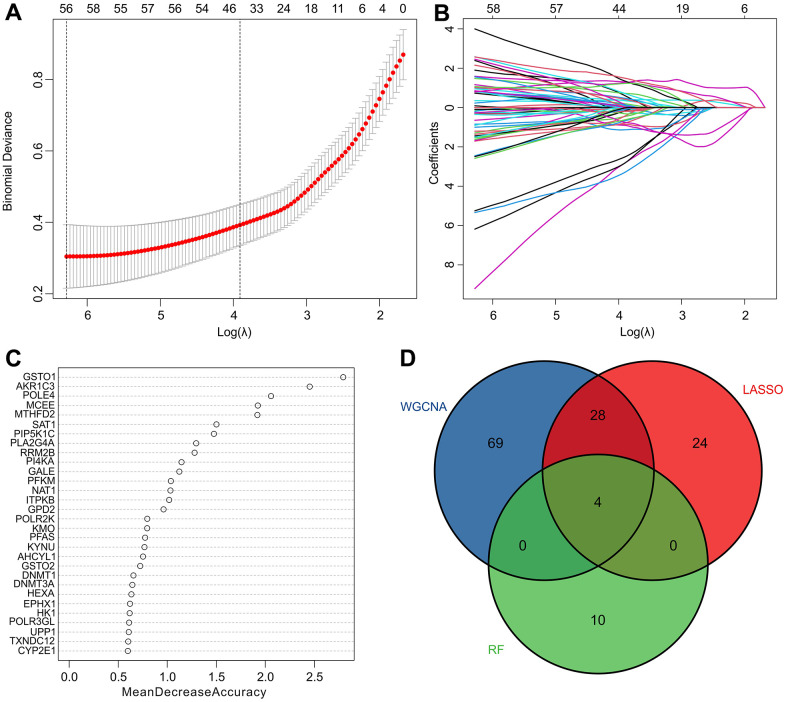
**Identification of diagnostic feature biomarkers via multiple machine learning algorithms.** (**A**, **B**) The least absolute shrinkage and selection operator (LASSO) algorithm shows the optimal coefficient based on the DE-MRGs. (**C**) The random forest (RF) algorithm shows the diagnostic feature biomarkers based on the DE-MRGs. (**D**) Venn diagrams show 4 diagnostic feature biomarkers using WGCNA, LASSO, and RF algorithms.

Several machine learning algorithms were employed to identify biomarkers with diagnostic potential. The least absolute shrinkage and selection operator (LASSO) algorithm identified 56 variables as diagnostic feature biomarkers for RA, as illustrated in Figure 4A, 4B. Additionally, the random forest (RF) algorithm identified 14 diagnostic biomarkers, as shown in Figure 4C. Through the application of WGCNA, LASSO, and RF algorithms, four diagnostic biomarkers were identified, including aldo-ketoreductase family 1 member C3 (AKR1C3), methylmalonyl-CoA epimerase (MCEE), and DNA polymerase 4 (POLE4), which can be observed in Figure 4D.

### Evaluation of diagnostic biomarkers effectiveness

**Figure 5:**
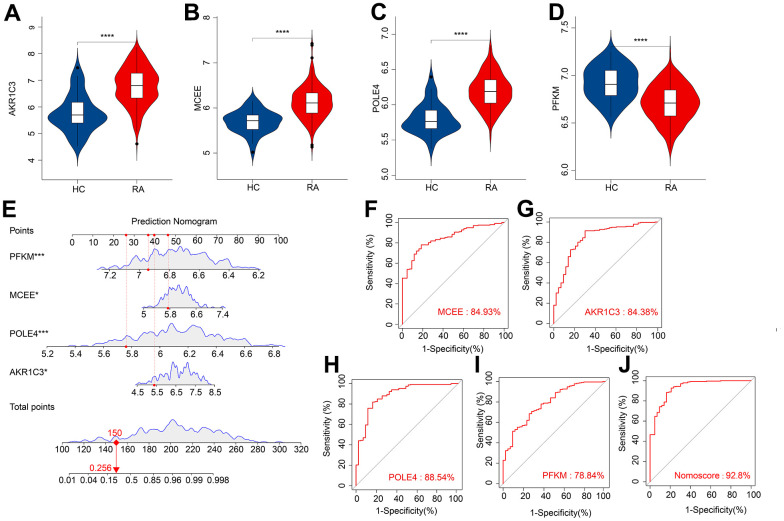
**ROC curve analysis and the expression of the diagnostic feature biomarkers.** The violin diagram shows the expression of (**A**) AKR1C3, (**B**) MCEE, (**C**) POLE4, and (**D**) PFKM. (**E**) A nomogram model to validate the impact of the four feature biomarkers on diagnostic effectiveness. ROC curve analysis of (**F**) MCEE, (**G**) AKR1C3, (**H**) POLE4 and (**I**) PFKM. (**J**) The AUC value of the nomogram score was 92.8%. *P < 0.05; **P < 0.01; ***P < 0.001; **** P < 0.0001.

**Figure 6:**
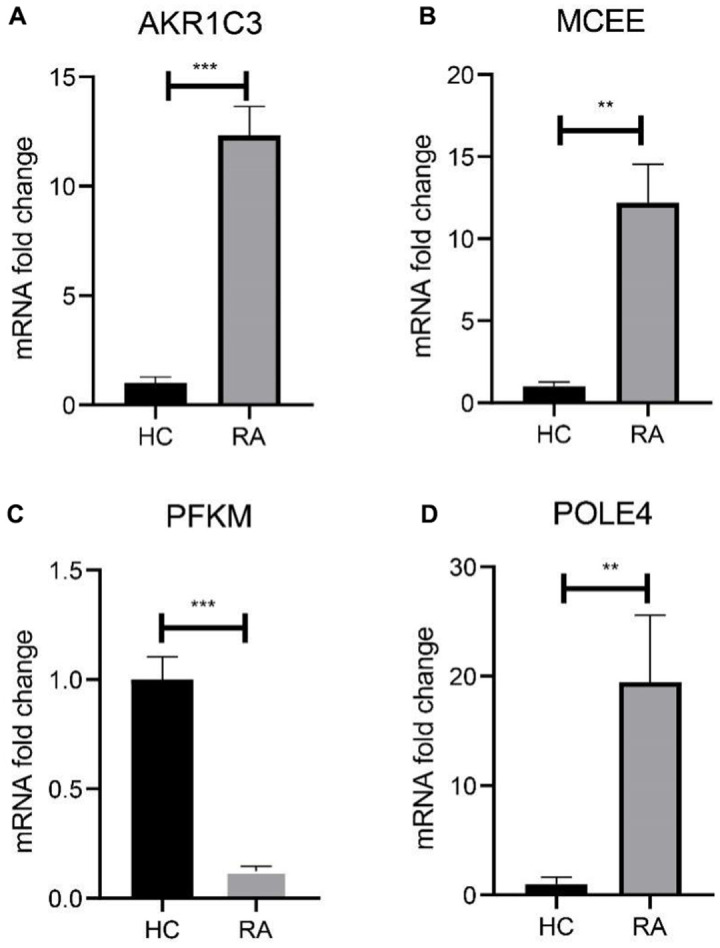
**Validation of 4 diagnostic feature biomarkers in clinical tissues via qRT-PCR.** The expression of (**A**) AKR1C3, (**B**) MCEE, (**C**) PFKM and (**D**) POLE4. Statistical significance: *P < 0.05; **P < 0.01; ***P < 0.001. ns: no significance.

**Figure 7:**
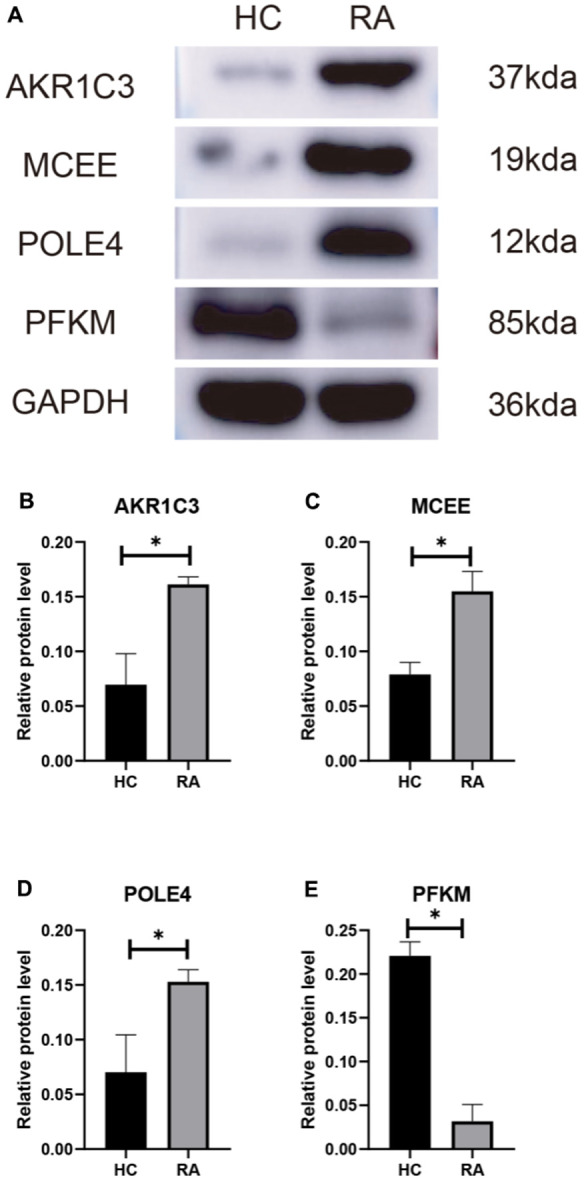
(**A**) Validation of 4 diagnostic feature biomarkers in clinical tissues via western blot analysis. The expression of (**B**) AKR1C3, (**C**) MCEE, (**D**) POLE4 and (**E**) PFKM. Statistical significance: *P < 0.05; **P < 0.01; ***P < 0.001. ns: no significance.

We subsequently investigated the expression and diagnostic effectiveness of the four previously identified diagnostic feature biomarkers for RA. AKR1C3, which has been reported to act as a regulator for hormone activity and prostaglandin F (PGF) synthase associated with promoting the release of inflammatory factors, was found to have a higher expression in the RA group in comparison with HC group (Figure 5A). This observation suggests that inflammation levels may be higher in the RA group. Furthermore, MCEE and POLE4 were upregulated significantly in RA patients compared to that in the HC group, while PFKM was downregulated in the RA group (Figure 5B–5D). A nomogram model was developed to validate the impact of the four feature biomarkers on diagnostic effectiveness (Figure 5E). The ROC curve analysis showed that the AUC values of AKR1C3, MCEE, POLE4, and PFKM were 84.38%, 84.93%, 88.54%, and 78.84%, respectively. The AUC value of the nomogram score was 92.8%, indicating its promising diagnostic effectiveness (Figure 5F–5J). Additionally, the expressions of the feature biomarkers were validated in clinical tissues, which demonstrated that patients with RA had a higher expression of AKR1C3, MCEE, and POLE4 and a lower expression of PFKM (Figures 6A–6D, 7A–7E). Our results showed that four diagnostic signature biomarkers have high diagnostic value for RA.

### Building CIA mouse model and validating diagnostic biomarkers

**Figure 8:**
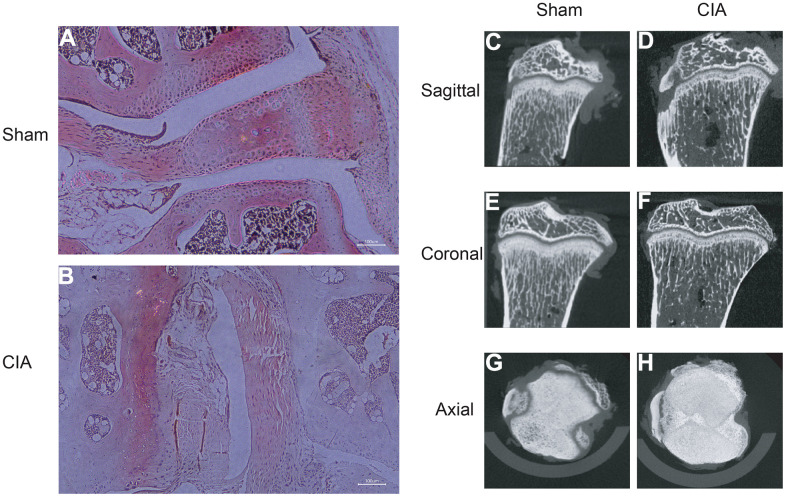
**HE staining of mouse joints and micro-CT imaging of mouse joints.** (**A**) HE staining of joints in the Sham group mice. (**B**) HE staining of joints in the CIA group mice. (**C**–**H**) Micro-CT imaging of joints in the Sham and CIA group mice.

**Figure 9:**
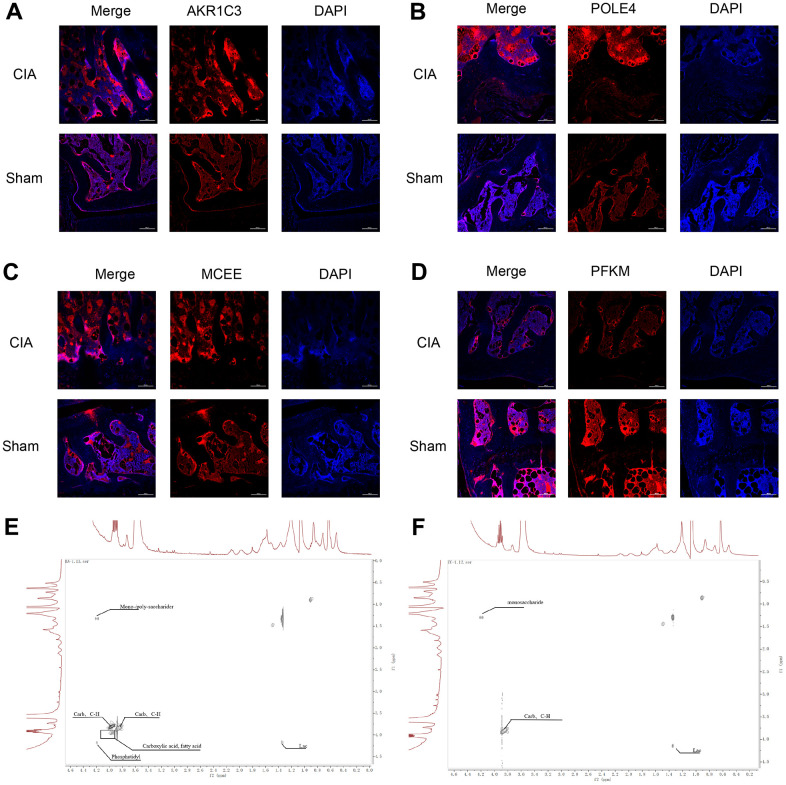
**Immunofluorescence staining of AKR1C3, POLE4, MCEE, and PFKM in mouse knee joints and NMR 2D Tocsy spectra of human meniscus tissue.** (**A**) Immunofluorescence staining of AKR1C3 in both groups of mice. (**B**) Immunofluorescence staining of POLE4 in both groups of mice. (**C**) Immunofluorescence staining of MCEE in both groups of mice. (**D**) Immunofluorescence staining of PFKM in both groups of mice. (**E**) NMR 2D Tocsy spectrum of meniscus tissue from RA patients. (**F**) NMR 2D Tocsy spectrum of normal meniscus tissue.

To verify the expression of the diagnostic biomarkers AKR1C3, MCEE, POLE4, and PFKM in RA, we developed a CIA mouse model. We obtained and stained knee joint sections of two successfully modeled groups of mice, and found that in comparison with the Sham group mice (Figure 8A), the histological analysis of the knee joint sections of CIA mice (Figure 8B) showed a significant up-regulation in the level of inflammation and immune cell infiltration, with crushed cartilage boundary. Using bone structure micro-CT, we scanned and detected the knee joints of both groups of mice. Sham group mice showed a smooth bone surface and relatively intact joint structure (Figure 8C, 8E, 8G), while CIA group mice showed bone and cartilage erosion, irregular bone surface structure, and severe osteoporosis (Figure 8D, 8F, 8H). These results confirm the successful modeling of RA in CIA mice. Furthermore, immunofluorescence results of the knee joints of the two groups of mice showed that AKR1C3, MCEE, and POLE4 were highly expressed in the CIA group mice compared to the Sham group, while PFKM was expressed at a lower level (Figure 9A–9D). This result is similar to what we obtained from clinical tissues. These results further demonstrate the high value of these four diagnostic biomarkers in the research of RA diagnosis and treatment.

### Human meniscus *ex vivo* tissue NMR 2D TOCSY spectra

The TOCSY spectra obtained from the rheumatoid arthritis group revealed a greater number of dispersed peaks with a hybridization of Carb and C-H compound peaks, which are associated with cartilage collagen metabolism. Additionally, there was a decrease in the molecular weight of the sugar peaks that are related to proteoglycan metabolism. The density of the Phosphatidyl peak group, which is associated with cell membrane and phospholipid metabolism, also increased with a more dispersed compound distribution. The accumulation of lipids and acidic substances was more pronounced, and the diversity of phosphoric acid metabolites increased. Furthermore, the variety of intermediate carbon metabolism substances increased, indicating a higher diversity of carbon metabolism. Overall, the complexity of the compounds was greater, and the peak distribution was of significant difference from that observed in normal cartilage tissue (Figure 9E, 9F).

### Immune infiltration landscape analysis

**Figure 10:**
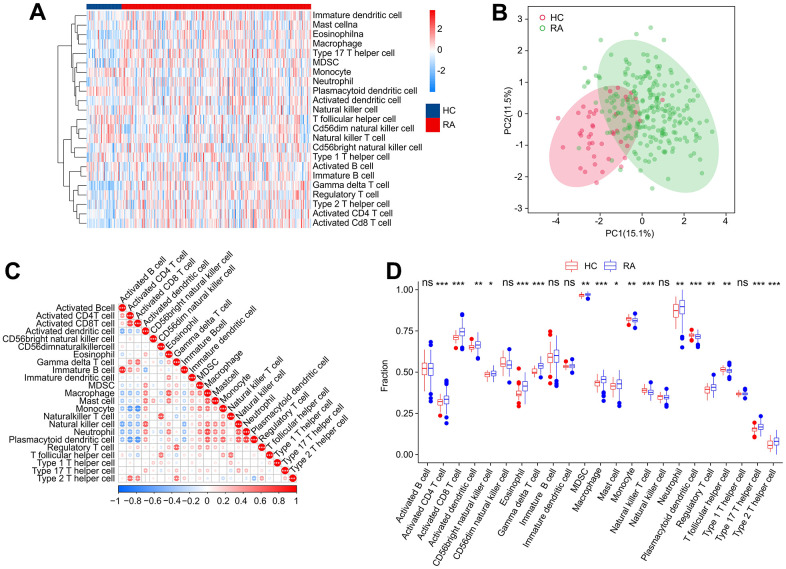
**Immune infiltration analysis.** (**A**) Heatmap shows the fraction of 23 types of immune cells in HC and RA groups based on the ssGSEA algorithm. (**B**) Principal component analysis (PCA) reveals a significant difference based on the 23 immune cells. (**C**) Correlation analysis of 23 immune cells. (**D**) The quantitative results of 23-type immune cells. Statistical significance: *P < 0.05; **P < 0.01; ***P < 0.001. ns: no significance.

Concerning RA is a characterized by a series of immune and inflammatory changes in its pathogenesis, we aimed to systematically investigate the differences in the immune infiltration landscape between HC and RA groups. Using ssGSEA algorithm, the study calculated 23 types of immune cells with the observation that the RA group showed a higher immune status than the HC group (Figure 10A). Principal component analysis (PCA) observed distinct clusters based on the component of 23 immune cells (Figure 10B). A significant correlation in most immune cells was revealed by the correlation analysis (Figure 10C). Specifically, the activity of B cells was positively correlated with immature B cells (r = 0.83, p < 0.001) but correlated with activated dendritic cells and natural killer cells negatively (r = -0.50, p < 0.001, r = -0.54, p < 0.001, respectively). CD4+ T cells were negatively correlated with plasmacytoid dendritic cells (r = -0.63, p < 0.001) and monocytes (r = -0.60, p < 0.001) but correlated with CD8+ T cells positively (r = 0.72, p < 0.001). Moreover, macrophages were observed to be positively correlated with eosinophils (r = 0.64, p < 0.001), mast cells (r = 0.62, p < 0.001), and neutrophils (r = 0.69, p < 0.001), while CD8+ T cells were inversely correlated with plasmacytoid dendritic cells (r = -0.67, p < 0.001) and monocytes (r = -0.68, p < 0.001). The quantitative results indicated that the proportion of CD4+ T cells, CD8+ T + cells, activated dendritic cells, CD56 bright natural killer cells, eosinophils, MDSC, macrophages, gamma delta T cells, mast cells, neutrophils, regulatory T cells and type 17/12 T helper cells were elevated remarkably in the RA group in comparison with the HC group. In addition, RA group had the lower proportions of monocyte, plasmacytoid dendritic cell, natural killer T cell and T follicular helper cell (Figure 10D). These results provide a clue to the role of the abnormal immune microenvironment in the regulation of RA development.

### Consensus clustering analysis

**Figure 11:**
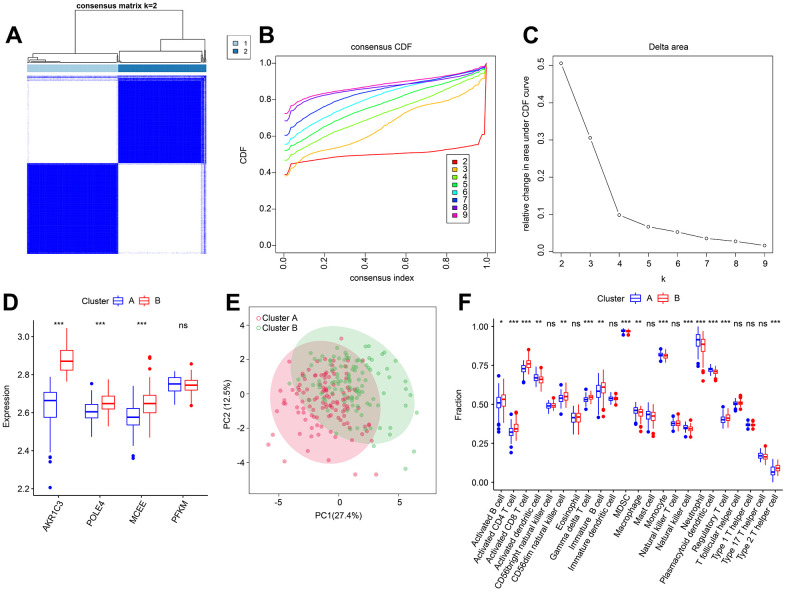
**Consensus clustering analysis of RA samples based on 4 diagnostic feature biomarkers.** (**A**) The consensus clustering heatmap shows the optimal K classification = 2-9. (**B**) Consensus CDF. (**C**) Delta area. (**D**) Expression of 4 diagnostic feature biomarkers in Cluster A and Cluster B. (**E**) PCA score plot. (**F**) The proportion of 23-types immune cells of patients with RA in Cluster A and B.

Based on the four diagnostic biomarkers, consensus cluster analysis was subsequently performed in order to classify RA samples into different molecular subtypes. The heatmap showed an optimal classification of RA samples at K = 2, with the number of 118 and 114 samples in Cluster A and B respectively (Figure 11A–11C). The expression of the four diagnostic biomarkers demonstrated that RA patients in Cluster A exhibited lower expression of AKR1C3, POLE4, and MCEE than those in Cluster B (Figure 11D). The result of PCA score plot displayed distinct clusters for RA patients in 23-type immune cells (Figure 11E). The ssGSEA result indicated that patients in Cluster B had relatively high proportions of the most of the 23-type immune cells (Figure 11F). Our results show that these four diagnostic biomarkers can accurately cluster RA samples into distinct molecular subtypes that are significantly associated with immunoinfiltrating landscapes.

### Correlation analyses between diagnostic feature biomarkers and immune infiltration landscape

**Figure 12:**
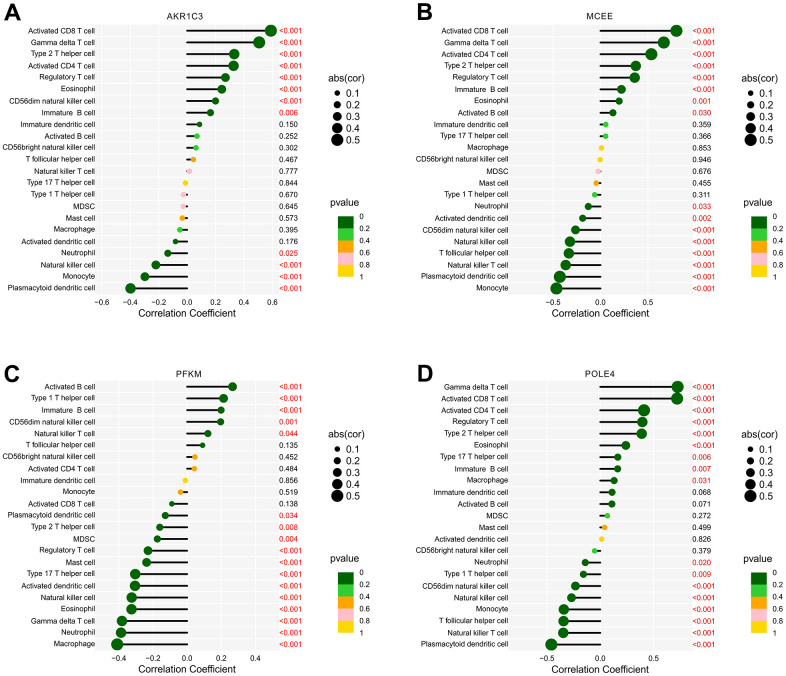
**Correlation analysis of diagnostic feature biomarkers and immune infiltration cells.** The lollipop diagram illustrates the correlation of 23 immune cells and (**A**) AKR1C3, (**B**) MCEE, (**C**) PFKM, and (**D**) POLE4. The dot represents the absolute value of the correlation coefficient, and the p -values are annotated with different colors. p < 0.05 is considered statistically significant.

Prior investigations have demonstrated robust associations between metabolic regulation and the function of the immune microenvironment. In light of this, our aim was to examine the relationship between the selected biomarkers and the level of immune infiltration landscape (Figure 12). Our correlation analysis revealed significant associations between four feature biomarkers and the level of immune cells. Specifically, AKR1C3 was correlated with CD8+ T cells and gamma delta T cells positively (r = 0.59, p < 0.001, r = 0.51, p < 0.001, respectively), while inversely correlated with the level of plasmacytoid dendritic cells (r = -0.4, p < 0.001; Figure 12A). MCEE was correlated positively with CD8+ T cells, CD4+ T cells and gamma delta T cells (r = 0.8, p < 0.001, r = 0.54, p < 0.001, r = 0.67, p < 0.001, respectively), but correlated with plasmacytoid dendritic cells and monocytes the opposite way (r = -0.43, p < 0.001, r = -0.46, p < 0.001, respectively; Figure 12B). PFKM had a negative correlation with macrophages (r = -0.41, p < 0.001; Figure 12C). Notably, POLE4 was positively correlated with gamma delta T cells, CD8+ T cells and CD4+ T cells (r = 0.73, p < 0.001, r = 0.72, p < 0.001, r = 0.41, p < 0.001, respectively), while inversely correlated with plasmacytoid dendritic cells (r = -0.46, p < 0.001; Figure 12D). Our findings indicate the correlation between four diagnostic biomarkers and immune infiltration landscape, which provides a potential molecular mechanism of immune microenvironment regulation in RA.

## DISCUSSION

RA leads to irreversible damage of the synovial lining of joints and significantly affects the life quality [18]. Over the past few decades, despite advances in therapeutic strategies, the prevalence of RA has increased [19]. Thus, it is crucial to understand the disease pathogenesis and develop effective treatment strategies. Recent studies have highlighted the role of abnormal metabolic programming and metabolism-mediated immunometabolism, involving glucose, energy, and lipid metabolism, in the development of RA [20–22]. Dynamic metabolic regulation affects the immune status of RA by altering the immune microenvironment [23]. Despite this, the contribution of MRGs to the onset and development of RA has yet to be determined. Aims of this study were to investigate the relationship between MRGs and immune infiltrates and to identify novel biomarkers for the diagnosis and treatment of RA. Four diagnostic feature biomarkers for RA were identified and validated through multiple machine-learning algorithms and *in vitro* experiments. The immune microenvironments significantly differed in terms of the expression of these four diagnostic feature biomarkers.

In this study, differential expressions of MRGs between the HC and RA groups were employed by multiple bioinformatics methods. GO enrichment analysis demonstrated that the DE-MRGs were significantly enriched in the biological process of small molecule catabolic processes, phospholipid metabolic processes, and ribose phosphate metabolic processes. The KEGG signaling pathway results suggested that purine metabolism, carbon metabolism, and inositol phosphate metabolism may mediate the contribution of DE-MRGs to the pathogenesis of RA. Purine is a key molecule in intracellular and extracellular signaling in nucleic acids, and its metabolism is associated with various diseases, including diabetes, cardiovascular diseases, and cancer [24–26]. The folate cofactor regulates carbon metabolism and is linked to several physiological processes, including purine and thymidine biosynthesis, redox defense and amino acid homeostasis [27, 28]. Carbon metabolism has been reported to affect the process of inflammation and is associated with an increased tumorigenesis risk [29]. Abnormal inositol phosphate metabolism, which is involved in energy metabolism and metabolic disorders, has been associated with multiple diseases [30]. In conclusion, our findings suggest several potential roles of MRGs in the development of RA.

Based on three machine learning algorithms, AKR1C3, MCEE, POLE4, and PFKM were identified as four diagnostic feature biomarkers. The ROC results demonstrated significant diagnostic value of these four biomarkers for RA. Furthermore, the results of clinical tissue and mouse model validation showed significant differences in the expression of these four diagnostic biomarkers in HC and RA tissues. The TOCSY two-dimensional spectra of metabolites revealed that the types of carbon compounds were significantly higher in RA patients compared to the normal group. Moreover, the study found that the types of phosphates and fatty acid substances were significantly increased in RA patients, indicating that RA patients had higher activity in purine metabolism, hormone level regulation, and energy metabolism related to carbon metabolism. These findings are consistent with the metabolic characteristics of the four diagnostic feature biomarkers. Aldo-ketoreductase family 1 member C3 (AKR1C3) is a member of aldoketone reductase superfamily [31]. As an NADP(H) oxidoreductase, AKR1C3 is a potential therapeutic target for various malignant tumors and endocrine diseases [32]. AKR1C3 catalyzes androgen, estrogen, progesterone and prostaglandin metabolism [33]. Sex hormones influence pathogenesis process by regulating immune cell activity and the sensitivity to immune-mediated damage, including the development of RA [34]. Men with RA tend to have lower serum androgen levels, and the incidence of RA increases as androgen production declines with age [35, 36]. The up-regulation of AKR1C3 we found in RA could be explained by regulating sex hormone-related pathways.

The role of MCEE in RA has not been thoroughly studied. MCEE metabolizes propionyl-coA into succinyl-CoA and methylmalonyl-CoA mutase (MUT) in the propionic acid catabolic pathway, which then enters the citric acid cycle [37]. Propyl coA is a common degradation product of branched amino acids and odd-chain fatty acids. Its accumulation inhibits n-acetyl glutamate synthase, an enzyme essential for maintaining the urea cycle [38]. Furthermore, the relationship between the inflammatory state and urea cycle activity has been investigated [38, 39], suggesting a potential mechanism for MCEE to participate in validating reactions in RA.

We observed a significant increase in POLE4 in the RA group. However, there are few reports about POLE4. As an important component of the lead polymerase Polε, POLE4 is closely associated with chromatin integrity during DNA replication [40]. Moreover, POLE4-deficient mice have been reported to have severe developmental abnormalities, T/B lymphocytopenia, and lymphoma formation [41]. Abnormal self-activation of T/B lymphocytes is an important part of RA development, and its treatment has been widely used in clinical practice [42]. The relationship between elevated POLE4 in RA and lymphocyte function requires further investigation.

Apart from the well-established immunological associations, the majority of the RA biomarkers selected in this study showed a positive correlation with γδ T cells. As a non-conventional T cell population, γδ T cells mediate recognition of non-peptide molecules directly, thereby playing a unique role in supporting immune progress [43]. Although the alteration of γδ T cell levels is not consistently reported in the peripheral blood of RA patients [44], some studies indicated that γδ T cells in the synovium of RA exhibit an activated phenotype with decreased CD16 expression and increased HLA-DR expression [45]. Moreover, the selective amplification of γδ T cells in synovial lymphocytes suggests a specific recognition by specific antigens in the synovium [46]. Functionally, γδ T cells was reported to exhibit antigen presentation, contribute to antibody production and predominantly express a Th1-like cytokine profile in RA patients [47]. In addition, γδ T cells have the potential to secrete multiple cytokines, including TNFα, and mediate effective cytotoxicity via type I and type II cytokines. Thus, γδ T cells may have the potential for immunomodulatory and even tissue metabolism regulation, which has recently become a focus of research [48]. The highly expressed γδ T cells in RA suggests their important role and may provide new insights into its pathogenesis.

Although our study has demonstrated a correlation between biomarkers and immune infiltration, it is important to acknowledge its limitations. Further experimental validation is needed to establish a causal relationship between the biomarkers and immune infiltration. In addition, confirmatory tests performed lack validation of large sample sizes of RA patients, so the specific role of these biomarkers in the development of RA remains to be further explored. Therefore, further research is needed to verify our findings and to better understand RA pathogenesis.

## CONCLUSIONS

In conclusion, our study utilizing a machine learning algorithm has identified AKR1C3, MCEE, POLE4, and PFKM as RA diagnostic feature biomarkers associating with immune infiltration. These findings provide a new perspective for RA development.
